# Lipoprotein(a) and Lung Function Are Associated in Older Adults: Longitudinal and Cross-Sectional Analyses

**DOI:** 10.3390/biomedicines12071502

**Published:** 2024-07-06

**Authors:** Chae Kyung Song, Olena Ohlei, Theresa Keller, Vera Regitz-Zagrosek, Sarah Toepfer, Elisabeth Steinhagen-Thiessen, Lars Bertram, Nikolaus Buchmann, Ilja Demuth

**Affiliations:** 1 Charité–Universitätsmedizin Berlin, Corporate Member of Freie Universität Berlin and Humboldt-Universität zu Berlin, Department of Endocrinology and Metabolic Diseases (Including Division of Lipid Metabolism), Augustenburger Platz 1, 13353 Berlin, Germany; 2 Lübeck Interdisciplinary Platform for Genome Analytics (LIGA), University of Lübeck, 23562 Lübeck, Germany; 3 Institute of Biometry and Clinical Epidemiology, Charité–Universitätsmedizin Berlin, Corporate Member of Freie Universität Berlin, Humboldt-Universität zu Berlin, Berlin Institute of Health, Reinhardtstraße 58, 10117 Berlin, Germany; 4 Institute for Gender in Medicine, Center for Cardiovascular Research, Charité–Universitätsmedizin Berlin, Corporate Member of Freie Universität Berlin, Humboldt-Universität zu Berlin, Berlin Institute of Health, 10099 Berlin, Germany; 5 Department of Cardiology, University Hospital Zürich, University of Zürich, 8057 Zürich, Switzerland; 6 Department of Cardiology, Charité–University Medicine Berlin, Campus Benjamin Franklin, 10117 Berlin, Germany; 7 Charité–Universitätsmedizin Berlin, BCRT-Berlin Institute of Health Center for Regenerative Therapies, 10117 Berlin, Germany

**Keywords:** lipoprotein(a), pulmonary health, spirometry, longitudinal follow-up, aging, sex difference, genetics

## Abstract

While numerous studies have confirmed a causal association between lipoprotein(a) [Lp(a)] and cardiovascular diseases, only a few studies have assessed the relationship between Lp(a) and pulmonary health, with inconsistent findings regarding this topic. This study’s aim was to examine whether levels of serum Lp(a) are associated with lung function in a dataset of relatively healthy older adults. We used longitudinal data collected at two time points 7.4 ± 1.5 years apart from 679 participants (52% women, 68 [65–71] years old) from the Berlin Aging Study II (BASE-II). Multiple linear regression models adjusting for covariates were applied to examine the association between Lp(a) and lung function. The forced expiratory volume in one second (FEV1) and the forced vital capacity (FVC) were higher in both men and women with higher Lp(a) levels. However, since this association between lung function parameters and Lp(a) was not supported by Mendelian randomization analyses using recent genome-wide association study data, these relationships should be investigated in future work, as the observed differences are, in part, considerable and potentially clinically relevant.

## 1. Introduction

Discovered by the Norwegian physician Kare Berg in 1963, lipoprotein(a) [Lp(a)] is composed of an LDL-like particle with an apolipoprotein B100 component covalently bound to an apolipoprotein(a) particle [1,2,3,4,5,6,7,8]. Over the last decade, numerous clinical data have shown that increased serum Lp(a) concentrations are associated with a higher risk of cardiovascular disease (CVD) [9,10,11,12]. While lifestyle factors, such as diet, physical activity, and smoking, can modify the risk of CVD, they do not significantly affect Lp(a) levels [13,14,15]. Mendelian randomizations and large epidemiological studies have accumulated strong evidence for Lp(a) acting as the single most common, independent, genetically inherited causal risk factor for CVD [16,17,18]. These compelling and consistent findings regarding the stark association between Lp(a) and cardiovascular events have generated high interest amongst researchers and physicians in understanding the pathogenicity of Lp(a) and discovering new lipid-lowering agents specifically targeted to lower Lp(a) concentration [19,20,21,22,23]. Currently, several drugs that specifically lower Lp(a), mostly by using RNA-targeting strategies, are under investigation in clinical trials. These drugs are able to lower plasma Lp(a) by up to 98%, with few side effects to date [24]. Whether this drug-based reduction in Lp(a) also leads to the anticipated reduction in CVD outcomes remains to be seen in the ongoing clinical studies. However, the physiological function of Lp(a) remains, for the most part, unclear, especially regarding its role in pulmonary health.

Impaired lung function is known to be associated with coronary artery disease, cardiovascular disease, stroke, and cardiovascular mortality, as well as all-cause mortality independent of cardiac function and other cardiovascular risk factors [25]. The close interrelationship between pulmonary and cardiovascular pathophysiology [26,27,28,29] raises the question of whether Lp(a) could also be a clinical risk factor for pulmonary health.

There have only been two studies so far that have investigated the direct relationship between Lp(a) and pulmonary health in the general population [30,31]. Both studies utilized spirometry to assess general lung health. Spirometry is a test that measures, among other parameters, the forced expiratory volume in one second (FEV1) and the forced vital capacity (FVC), and is the most common pulmonary function test used to detect and monitor chronic lung diseases [32]. According to our previous analyses of cross-sectional data from the Berlin Aging Study II (BASE-II) (n = 606, 55.1% females, 68 [60–84] years old), high serum Lp(a) levels were associated with higher FEV1 in older men [30]. Conversely, a cross-sectional study by Lee et al. in 2017 (n = 64,082, 48.4% women, 38 ± 7 years old) showed that a high Lp(a) level was associated with a lower FEV1, as well as a lower FVC in a large study population consisting of Korean health-screening subjects [31]. As a result of these inconsistent findings and a clear lack of other studies, the relationship between Lp(a) and lung function needs to be further investigated.

Therefore, in the present study, we conducted a follow-up analysis using Lp(a) concentrations measured at the baseline in the BASE-II [33], and newly measured spirometry measurements which were assessed for the BASE-II participants as part of the GendAge study [34]. Thus, the aim of the current study was to determine whether the Lp(a) serum levels assessed at the baseline were also longitudinally (mean follow-up of 7.4 years) associated with lung function, as observed in our previously reported cross-sectional analyses. To further investigate the implied relationship between Lp(a) and lung function, particularly with respect to causality, we conducted Mendelian randomization (MR) analyses combining recent, large-scale, genome-wide association study (GWAS) data for both outcomes.

## 2. Materials and Methods

### 2.1. Study Participants

The data analyzed in the current study were collected from the participants of the Berlin Aging Study II (BASE-II), including follow-up data which were assessed as part of the GendAge study 7.4 ± 1.5 years later [33,34]. Launched in 2009, BASE-II comprises a convenience sample and the study aimed to investigate the factors of “healthy” versus “unhealthy” aging in residents of greater Berlin, Germany. In order to understand the complex and multifaceted aspects of aging, this multidisciplinary study encompassed a wide range of disciplines, such as geriatrics, immunology, psychology, genetics, biology, sociology, as well as economics. A wide variety of data were collected from a cohort of 1600 older subjects aged 60 to 80 years, and a control group of 600 younger adults aged 20 to 35 years, which was not considered in the current analysis. The cross-sectional baseline examination was completed in 2014 [33]. In total, 1,083 BASE-II participants from the older group were medically re-examined between June 2018 and March 2020 as part of the GendAge study [34]. We additionally considered 17 participants who completed assessments in the GendAge study, whose medical baseline data were not available (these 17 participants were part of the BASE-II sample and were examined, at least, at one of the other BASE-II study sites). The cohort analyzed in the current study is larger than, and does not completely overlap with the cohort in the study by Buchmann et al. [30]. A higher number of spirometry measurements with a quality grade of “C” or above were available from the follow-up assessments compared to the baseline dataset; thus, we were able to include a higher number of subjects in the current analysis. All the participants gave written informed consent and the Ethics Committee of the Charité–Universitätsmedizin Berlin approved this study in 2009 and 2016 (approval numbers EA2/029/09 and EA2/144/16). This study was carried out in accordance with the latest revision of the Declaration of Helsinki and was registered with the German Clinical Trials Registry as DRKS00009277 and DRKS00016157.

### 2.2. Exclusion Criteria

**Figure 1:**
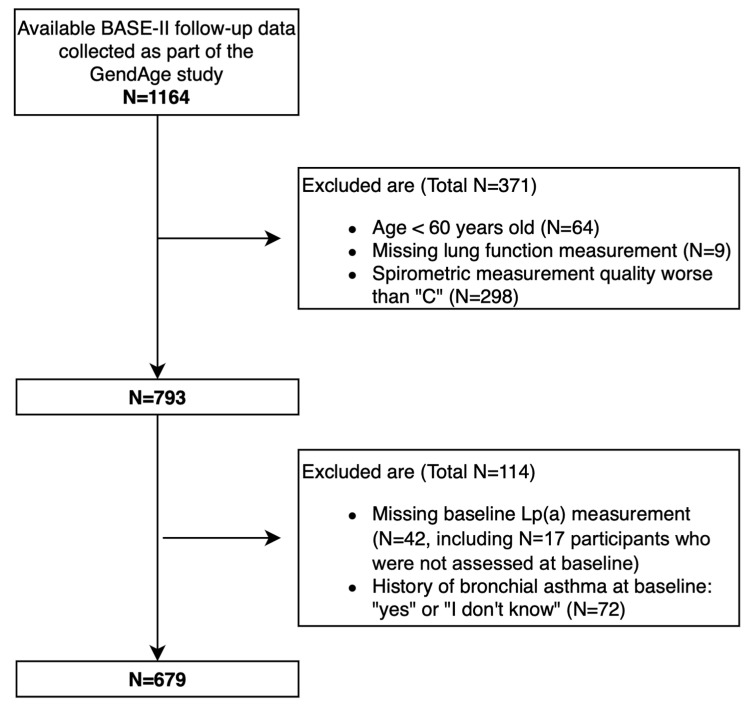
Flow chart of included and excluded study participants. A total of 1164 participants were included in the dataset from the follow-up study, which also included participants who had participated in the baseline study. After implementing the exclusion criteria as listed above, 485 participants were excluded, leaving a total of 679 participants to be analyzed in the current study.

In accordance with the criteria from our previous analyses of the BASE-II, we excluded subjects who were younger than 60 years old at the baseline. In accordance with our previous study [30], those with a spirometry test quality grade lower than a “C”, and those who either answered “yes” or “I don’t know” regarding a self-reported history of bronchial asthma, were excluded. We further excluded subjects with missing Lp(a) data from the baseline and missing lung function measurements (i.e., FEV1 and FVC) from the follow-up assessment. This resulted in a total of 679 older subjects analyzed in the current study (Figure 1).

### 2.3. Lipoprotein(a) Measurement

Blood samples were extracted after >8 h of fasting and kept at 4–8 degrees Celsius until analyzed on the same day. The concentration of Lp(a) was measured using particle-enhanced immunological turbidity tests in a certified laboratory (Labor Berlin GmbH, Berlin, Germany). In the baseline assessment, Lp(a) was measured in mg/dL using a turbidimetric LPALX assay (Roche) on a cobas c 701/702 system, according to the recommendation of the manufacturer. The precipitate was quantified turbidimetrically at 450 nm. The assay was independent of apolipoprotein(a) isoform size. The detection limit was 3 mg/dL.

In the follow-up assessment, Lp(a) was measured in nmol/l by a particle-enhanced turbidimetric immunoassay with Tina-quant Lipoprotein(a) Gen.2 (Latex) (LPA2) Roche^®^ on a cobas system. The precipitate was quantified turbidimetrically at 800/660 nm. The detection limit was 7 nmol/L.

Lp(a) concentrations lower than the detection limit of 3 mg/dL or 7 nmol/L were recorded as 1.5 mg/dL or 3.5 nmol/L, respectively. For comparison purposes, we converted the Lp(a) values in nmol/l into mg/dL using the following conversion factor as recommended by the manufacturer mentioned above: mg/dL = (nmol/L + 3.83) × 0.4587.

### 2.4. Measurement of Pulmonary Function

Spirometry was performed as recommended by the American Thoracic Society using an EasyOne^TM^ Spirometer (nnd Medizintechnik AG, Zurich, Switzerland) [28]. A minimum of two spirometry measurements with a sufficient level of quality had to be available, with the difference in the two highest FEV1 and FVC values being less than or equal to 200 mL (categorized as quality grade A, B, or C) to be included in the current analysis. The highest FEV1 and FVC values from two or more tests with a sufficient level of quality were used for the analysis. All 679 participants had spirometry measurements of sufficient quality grade at follow-up. Out of this cohort, a subtotal of 356 participants had spirometry measurements with a quality grade of “C” or above from the baseline assessment. We investigated the difference in lung function between the baseline and follow-up (delta FEV1 and delta FVC) in relation to the Lp(a) concentrations measured at the baseline in these 356 participants. The same spirometer type was used for the baseline and follow-up assessments, and the quality grade was scored automatically via the spirometer.

### 2.5. Covariables

Body weight was measured with minimal clothing to the nearest 0.1 kg, and height was measured to the nearest 0.1 cm using an electronic weighing and measuring station (seca 764, seca, Hamburg, Germany), and the measurements were used to calculate the body mass index (BMI). Standardized questions were used to assess the following covariables: pack-years, smoking status, regular alcohol intake, and physical inactivity. Smoking status was categorized dichotomously as either currently smoking or currently not smoking, which included previous smokers. For alcohol intake, the participants were asked if they consumed alcohol regularly (either “yes” or “no”), and for the assessment of physical inactivity, the participants were asked if they were rarely or never physically active (either “yes” or “no”).

The comorbidities and morbidity burden of the participants were evaluated using a morbidity index (MI) largely based on the categories of the Charlson Comorbidity Index [35]. Multiple diagnoses were used to calculate the MI and these diagnoses were obtained mainly through an assessment of the medical history by the study physicians. The MI, ranging from 0 to 10, is a weighted sum of moderate-to-severe, mostly chronic illnesses, including but not limited to cardiovascular (e.g., congestive heart failure), cancer (e.g., lung cancer), pulmonary (e.g., chronic obstructive pulmonary disease, chronic bronchitis), and metabolic diseases (e.g., type 2 diabetes mellitus). HIV and peptic ulcer disease were not included in the calculation of the MI. The prevalence of type 2 diabetes mellitus (T2D) was determined based on the guidelines of the American Diabetes Association [36], considering a self-reported history of diabetes, laboratory measurements (fasting glucose and oral glucose tolerance test in participants with an unknown history of T2D), and consumption of diabetes-specific medication. Metabolic syndrome (MetS) was defined by using the criteria suggested by Alberti et al. [37].

### 2.6. Statistical Analysis

The Kolmogorov–Smirnov test was used to determine the normal distribution. Analogous to the previous cross-sectional study by Buchmann et al. [30], the baseline serum Lp(a) concentration values were divided into quintiles 1 to 5, and categorized into dichotomous groups—Lp(a) quintile 1 versus Lp(a) quintiles 2-5. The concentrations of Lp(a) in quintile 1 ranged from levels below the detection limit of <3 mg/dL to 3.4 mg/dL in men, and from <3 mg/dL to 4.0 mg/dL in women. The concentrations of Lp(a) in quintiles 2-5 ranged from 3.5 mg/dL to 176.6 mg/dL in men, and 4.1 mg/dL to 219.0 mg/dL in women. In multiple linear regression analyses, the Lp(a) quintile 1 and Lp(a) quintiles 2-5 were used as binary variables, in which Lp(a) quintile 1 was tested against Lp(a) quintiles 2-5. Model 1 was adjusted for age. In model 2, lifestyle factors (regular alcohol intake, self-reported physical inactivity, pack-years, and BMI) were added. In model 3, we additionally adjusted for morbidity (morbidity index). Due to the much broader range of Lp(a) concentrations in Lp(a) quintiles 2-5 compared to Lp(a) quintile 1, we used a one-way analysis of covariance (ANCOVA) to additionally investigate the individual quintiles separately, and to determine the differences in the adjusted means of lung function measurements for each Lp(a) quintile 1 through 5. Changes in the FEV1 and FVC measurements between the baseline and follow-up (delta FEV1 and delta FVC), in relation to Lp(a) concentrations at the baseline, were investigated using ANCOVA. This analysis included only those participants who had FEV1 and FVC measurements from both the baseline and follow-up, resulting in 356 participants. All the analyses in this study are explorative, resulting in *p*-values that are interpreted descriptively. These statistical analyses were carried out using IBM SPSS Statistics for Windows, Version 25.0 (IBM Corp., Armonk, NY, USA).

### 2.7. Mendelian Randomization Analyses

To investigate whether the association between Lp(a) and lung function parameters represent a causal relationship, we performed two-sample MR analyses using results from a recent large-scale GWAS on Lp(a) plasma levels and lung function. An MR is a type of instrumental variable analysis where the genetic variants (e.g., from the GWAS) of two traits (here, Lp(a) levels and FEV1, FVC) are combined to allow for inferences about a causal relationship between the traits [38]. To this end, we utilized summary statistics from recent GWAS results derived from UK biobank samples for both traits. For Lp(a), this included GWAS data generated by Sinnott-Armstrong et al. [39], who analyzed 35 blood traits in ~360 K individuals. For lung function, we utilized summary statistics from the GWAS by Shrine et al. [40], who investigated lung function parameters (including FEV1 and FVC) in ~590 K individuals. Two-sample MR was performed using the R package MendelianRandomization version 0.060 [41] running four analysis models: simple median, weighted median, inverse-variance weighted (IVW), and Egger regression. Each model makes different assumptions and uses different strategies to avoid false-positive causal inferences. Only the independent (r^2^ < 0.01, 1000 kb) single-nucleotide polymorphisms (SNPs) with a minor allele frequency (MAF) of >0.01 that showed a genome-wide significant association (i.e., *p*-values < 5 × 10^−8^) with Lp(a) in REF [39] were included in these analyses, after an outlier correction with MR-PRESSO [42].

## 3. Results

### 3.1. Descriptive Characteristics of Baseline Data and Lung Function Measurements at Follow-Up

The characteristics of the participants captured at the baseline and their lung function at the follow-up are summarized in Table 1. In the baseline examination, Lp(a) was measured in 1584 participants aged 60 years and older, and 679 of these participants underwent a successful lung function test at the follow-up, 7.4 ± 1.5 years later. At the baseline, the women had a higher median Lp(a) level (12.0 mg/dL [5.0–42.0] vs. 9.0 mg/dL [4.0–21.0], *p* = 0.002), a lower median BMI (25.1 kg/m^2^ [22.9–28.0] vs. 26.9 kg/m^2^ [24.8–29.0], *p* < 0.001), and lower median pack-years (0.0 years [0.0–6.1], vs. 7.0 years [0.0–22.2], *p* < 0.001) than the men. There was a higher proportion of participants with type 2 diabetes and metabolic syndrome for the men than for the women (*p* < 0.001). At the baseline, there were no statistically apparent differences between the men and women in terms of age, smoking status, regular alcohol intake, self-reported physical inactivity, or morbidity index (Table 1). As expected, the men had a higher mean FEV1 and FVC than the women, whereas the median FEV1/FVC was higher in the women (*p* < 0.001, Table 1).

### 3.2. Descriptive Characteristics Comparing Baseline and Follow-Up Data

**Figure 2:**
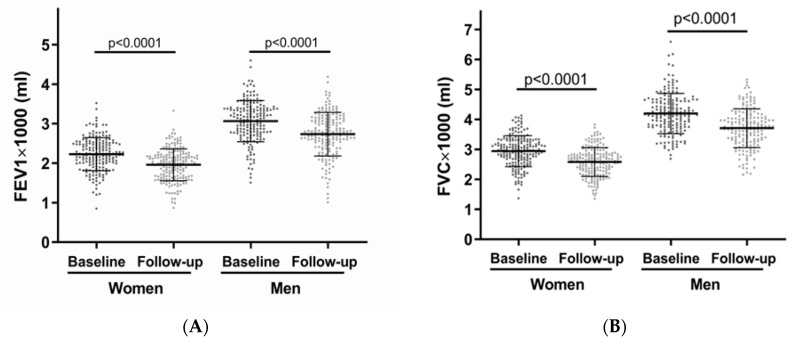
Paired comparison of lung function measurements at baseline vs. follow-up. Mean forced expiratory volume in 1 s (FEV1) (**A**) and mean forced vital capacity (FVC) (**B**) measured at baseline and follow-up are shown separately for men and women. Both mean FEV1 and FVC measurements declined after 7.4 ± 1.5 years in both sexes (paired *t*-test). Men showed higher mean FEV1 and FVC measurements compared to women.

Changes between the baseline and follow-up characteristics were determined by comparing the paired values from the two assessments (Table 2). For the men, the average BMI and self-reported physical inactivity did not change after 7.4 ± 1.5 years (*p* = 0.136 and *p* = 0.401, respectively), while for the women, the average BMI increased from 25.1 kg/m^2^ (22.9–28.0) to 25.5 kg/m^2^ (23.0–28.7) (*p* < 0.001). Self-reported physical inactivity increased among the women after 7.4 years (*p* = 0.028). The proportion of the men as well as the women currently smoking and drinking alcohol regularly decreased, and the morbidity index increased after 7.4 years. There was a significant increase in the number of subjects diagnosed with T2D at the follow-up in both the men and women (*p* < 0.001). There was no significant difference between the Lp(a) concentrations measured at the baseline and follow-up in both sexes. In the men, however, there was a change in the distribution of those categorized in Lp(a) quintile 1 after 7.4 ± 1.5 years (20% at the baseline vs. 25% at the follow-up with an Lp(a) below the detection limit, *p* = 0.010). In the women, this distribution did not change after 7.4 years. Furthermore, Table 3 and Figure 2 present the difference in the mean FEV1 and FVC between the baseline and follow-up, illustrating the decline in pulmonary function within the 7.4 years. A total of 356 participants had a paired set of lung function measurements from both the baseline and follow-up that also met the spirometry quality criteria. The men had a mean FEV1 of 3064 ± 521 mL at the baseline and 2733 ± 551 mL at the follow-up, and the women had a mean FEV1 of 2228 ± 418 mL at the baseline and 1958 ± 404 mL at the follow-up. As expected, the FEV1 and FVC decreased with age in both sexes (*p* < 0.001), while the FEV1/FVC ratio did not show a significant change over time.

### 3.3. Descriptive Characteristics According to Lp(a) Quintile 1 vs. Lp(a) Quintiles 2-5

**Figure 3:**
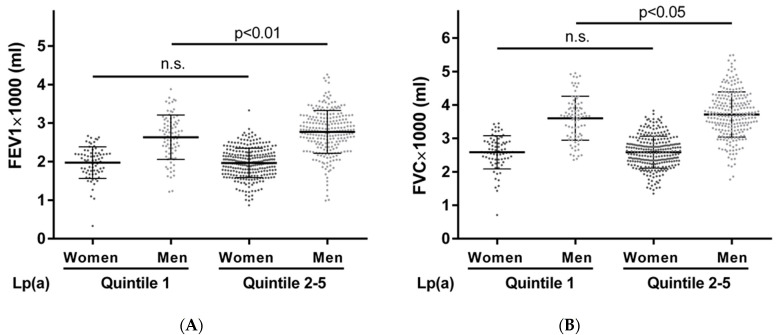
Longitudinal analysis of association between Lp(a) measured at baseline and lung function measured at follow-up, 7.4 years (mean) later. Distributions of FEV1 (**A**) and FVC (**B**) measurements are compared between participants belonging to Lp(a) quintile 1 and Lp(a) quintiles 2-5 separately for men and women. Mean FEV1 and FVC were higher in men categorized in Lp(a) quintiles 2-5 (Student’s *t*-test).

The characteristics of the participants captured at the baseline and their lung function at the follow-up, according to Lp(a) quintile 1 vs. Lp(a) quintiles 2-5, are summarized in Table 4. The men in Lp(a) quintiles 2-5 showed higher FEV1 and FVC measurements at the follow-up (2776 ± 555 mL and 3728 ± 676 mL, respectively), compared to those in Lp(a) quintile 1 (2577 ± 570 mL and 3520 ± 630 mL, respectively) (*p* = 0.010 and *p* = 0.026, respectively). The distributions of lung function measurements according to quintile 1 vs. quintiles 2-5 are illustrated in Figure 3. In the women, the mean FEV1 and FVC were marginally higher in Lp(a) quintiles 2-5 compared to those of the women in Lp(a) quintile 1. While these measurements showed a similar trend to those of the men, there was no statistical difference in lung function between quintile 1 vs. quintiles 2-5 in the women. A difference in the FEV1/FVC ratio between the two Lp(a) groups was not statistically apparent in both the men and women. As previously reported [43], we observed a higher proportion of participants with type 2 diabetes in Lp(a) quintile 1 (*p* = 0.010). Other baseline characteristics (i.e., age, BMI, smoking status, pack-years, regular alcohol intake, self-reported physical inactivity, and morbidity index) did not differ statistically between Lp(a) quintile 1 and Lp(a) quintiles 2-5.

### 3.4. Multiple Linear Regression Models Assessing the Association between Baseline Lp(a) and Follow-Up Lung Function Measurements

Multiple linear regression analyses with adjustments for baseline covariables were performed to assess whether Lp(a) quintile 1 is independently associated with lower lung function assessed 7.4 years later (Table 5). Model 1 was adjusted for age. In model 2, lifestyle factors, such as regular alcohol intake, self-reported physical inactivity, pack-years, and BMI, were added. The morbidity index was additionally adjusted for in model 3. In the men, very low concentrations of Lp(a) [Lp(a) quintile 1] were associated with lower levels of FEV1 and FVC for all three models. According to the highest-adjusted model 3, Lp(a) quintile 1 was associated with 218 mL and 234 mL lower levels of FEV1 (*p* = 0.008) and FVC (*p* = 0.017), respectively, in the men, when compared to these parameters in men with higher Lp(a) levels (quintiles 2-5). In the women, the baseline Lp(a) levels showed no association with lung function when assessed 7.4 years later. When the men and women were analyzed together (n = 679), Lp(a) quintile 1 was associated with lower FEV1s and FVCs for all three models (*p* = 0.006 for FEV1 and *p* = 0.009 for FVC, in model 3). The recalculation of models 1 through 3 using the covariables from the follow-up study further showed a statistically significant association between Lp(a) quintile 1 and lower FEV1 and FVC in the men, and no significant association in the women (Supplementary Table S1).

### 3.5. Adjusted Mean of FEV1 and FVC at Follow-Up According to Individual Lp(a) Quintile

**Figure 4:**
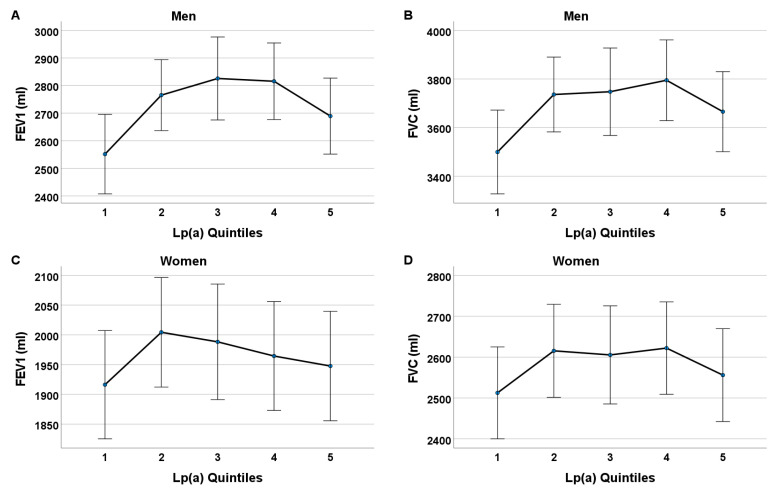
Adjusted means of FEV1 (**A**,**C**) and FVC (**B**,**D**) at follow-up according to each Lp(a) quintile 1 to 5 from baseline with 95% confidence interval (CI)—adjusted for covariates from baseline. One-way analysis of covariance (ANCOVA) was conducted to calculate and compare the mean FEV1 and FVC of each Lp(a) quintile 1 to 5 whilst controlling for covariates from baseline (i.e., age, BMI, alcohol intake, pack-years, self-reported physical inactivity, and morbidity index). In both men (**A**,**B**) and women (**C**,**D**), adjusted mean FEV1 and FVC for Lp(a) quintiles 2-5 were consistently higher than adjusted mean FEV1 and FVC for Lp(a) quintile 1. In men (**A**,**B**), those categorized in Lp(a) quintiles 3 and 4 showed highest adjusted mean FEV1 and FVC measurements, with an adjusted mean FEV1 of 2826 mL and 95% CI [2675, 2976] for quintile 3, and adjusted mean FVC of 3795 mL and 95% CI [3629, 3961] for quintile 4. Adjusted means of FEV1 and FVC in Lp(a) quintile 5 were slightly lower than those for Lp(a) quintiles 2-4, but remained consistently higher than those for Lp(a) quintile 1. In men, there were meaningful differences in adjusted means of FEV1 and FVC between Lp(a) quintile 1 vs. 2, quintile 1 vs. 3, and quintile 1 vs. 4 (*p* < 0.05). There was no meaningful difference in the adjusted means of FEV1 and FVC between Lp(a) quintile 1 and 5.

To further determine whether there were any differences in lung function measurements between each baseline Lp(a) quintile group, we ran a one-way analysis of covariance (ANCOVA) with age, BMI, alcohol intake, pack-years, self-reported physical inactivity, and morbidity index as the covariables. The estimated marginal adjusted means and 95% confidence intervals (95% CI [LL, UL]) of FEV1 and FVC, according to the individual Lp(a) quintiles 1 through 5, are illustrated in Figure 4. The participants in Lp(a) quintile 1 showed the lowest adjusted mean FEV1 and FVC measurements. The men in Lp(a) quintile 3 displayed the highest lung function measurements, with an adjusted mean FEV1 of 2826 mL and 95% CI [2675, 2976], and the men in quintile 4 displayed the highest adjusted mean FVC of 3795 mL and 95% CI [3629, 3961]. The adjusted means of FEV1 and FVC in Lp(a) quintile 5 were slightly lower than those of Lp(a) quintiles 2-4, but remained consistently higher than those of Lp(a) quintile 1. In the men, there were statistically evident differences in the adjusted means of FEV1 between Lp(a) quintile 1 vs. 2, quintile 1 vs. 3, and quintile 1 vs. 4 (*p* < 0.05). Likewise, the women from Lp(a) quintile 1 displayed the lowest mean lung function volumes compared to those from quintiles 2-5.

### 3.6. Delta FEV1 and Delta FVC According to Individual Lp(a) Quintile

**Figure 5:**
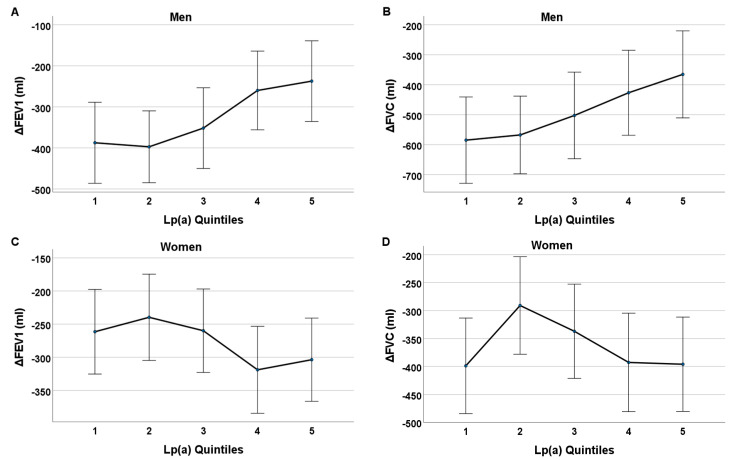
Adjusted means of delta FEV1 (**A**,**C**) and delta FVC (**B**,**D**) according to each Lp(a) quintile 1 to 5 from baseline with 95% confidence interval (CI)—adjusted for covariates from baseline. One-way analysis of covariance (ANCOVA) was conducted to calculate and compare mean differences in FEV1 and FVC from follow-up to baseline according to each Lp(a) quintile 1 to 5, whilst controlling for covariates from baseline (i.e., age, BMI, alcohol intake, pack-years, self-reported physical inactivity, morbidity index, and baseline FEV1 or FVC). In men, absolute delta FEV1 and absolute delta FVC were highest (i.e., decline in lung function volume was greatest after an average of 7.4 years) in those among lower Lp(a) quintiles. Difference in FEV1 was greatest in quintile 2 (−397 mL with 95% CI [−485, −310]) in men. Men in Lp(a) quintile 1 showed difference of −387 mL with 95% CI [−486, −289] in FEV1 and difference of −585 mL with 95% CI [−729, −441] in FVC. Meanwhile absolute delta FEV1 and delta FVC were both lowest in Lp(a) quintile 5 (i.e., decline in lung function volume was smallest after an average of 7.4 years). Men in Lp(a) quintile 5 showed difference of −237 mL with 95% CI [−335, −139] in FEV1 and difference of −365 mL with 95% CI [−511, −220] in FVC. Linear models in Figure 5 display consistent downward trend of delta FEV1 and FVC from Lp(a) quintile 1 to 5 in men. In women, no obvious relationship is shown.

We examined the mean difference in the FEV1 and FVC (delta FEV1 and delta FVC) between the baseline and follow-up according to the Lp(a) concentrations at the baseline. This analysis included those who additionally had FEV1 and FVC data available from the baseline with a sufficient quality grade, resulting in 356 participants (of the general cohort of 679 subjects in the main analysis). Here, we also adjusted for covariables from the baseline (age, regular alcohol intake, pack-years, self-reported physical inactivity, BMI, and morbidity index), as well as for the FEV1 or FVC measurements from the baseline. In the men, the absolute delta FEV1 and absolute delta FVC were greatest (i.e., the decline in lung function volume was greatest after an average of 7.4 years) in those among the lower Lp(a) quintiles. The difference in FEV1 was greatest in quintile 2 (−397 mL and 95% CI [−485, −310]) in the men. The men in Lp(a) quintile 1 showed a difference of −387 mL and 95% CI [−486, −289] in FEV1, and a difference of −585 mL and 95% CI [−729, −441] in FVC. Meanwhile, the absolute delta FEV1 and delta FVC were both lowest in Lp(a) quintile 5 (i.e., the decline in lung function volume was smallest after an average of 7.4 years). The men in Lp(a) quintile 5 showed a difference of −237 mL and 95% CI [−335, −139] in FEV1, and a difference of −365 mL and 95% CI [−511, −220] in FVC. The linear models in Figure 5 display a consistent upward trend of delta FEV1 and FVC from Lp(a) quintile 1 to 5 in the men. In the women, no obvious relationship is shown.

### 3.7. Cross-Sectional Analyses: Association between Re-Assessed Lp(a) and Lung Function Measurements at Follow-Up

The comparison between Lp(a) quintile 1 vs. Lp(a) quintiles 2-5, and multiple linear regression analyses were repeated with the cross-sectional follow-up data only, i.e., using serum Lp(a) values, lung function measurements, and the covariables that were re-assessed at the follow-up. The participant characteristics are shown in Supplementary Table S2. Without any adjustment, the relationship between Lp(a) and lung function was comparable to the longitudinal findings for the men. In the men, the mean FEV1 and FVC values were higher in Lp(a) quintiles 2-5 compared to those from Lp(a) quintile 1 (*p* = 0.055). The men in Lp(a) quintiles 2-5 showed a higher FEV1/FVC ratio compared to that of the men in Lp(a) quintile 1 (76% [71–79] vs. 74% [70–77], *p* = 0.035). The women showed no statistically meaningful difference in lung function measurements between the two Lp(a) groups (Supplementary Table S2). In the multiple linear regression analyses (Supplementary Table S3), model 1 shows that a lower Lp(a) level was associated with a lower FEV1 and FEV1/FVC when adjusted for age in the men (*p* = 0.038 and *p* = 0.048, respectively). When adjusted for further covariables (regular alcohol intake, self-reported physical inactivity, pack-years, BMI, and morbidity index), a lower Lp(a) level was associated with lower values of lung function measurements, but the association was no longer statistically apparent (models 2 and 3). The mean FEV1 and FVC, according to the two Lp(a) groups using the Lp(a) measured at the follow-up, are illustrated in Supplementary Figure S1.

### 3.8. Two-Sample MR Analyses Do Not Suggest a Causal Relationship between Lp(a) and FEV1/FVC

The MR analyses using recent UK biobank GWAS data did not suggest a causal relationship between Lp(a) and either FEV1 or FVC (Supplementary Figure S2). The results were consistent across both traits and all the MR models used. Given the very large sample sizes of the primary GWAS, these MR analyses were sufficiently powered to detect causal effects if they existed.

## 4. Discussion

In this study, we employed a longitudinal dataset of 679 older community-dwelling participants from the BASE-II to analyze the association between serum Lp(a) concentrations at the baseline and lung function measured at the follow-up, on average 7.4 years later. We further analyzed the decline in lung function in relation to the Lp(a) levels at the baseline in a sub-cohort of subjects who underwent spirometry measurements both at the baseline and follow-up. The main finding of the current study is that a higher baseline Lp(a) level is associated with better pulmonary function as assessed by FEV1 and FVC, on average, 7.4 years later. While showing a similar pattern in the men and women, statistically significant differences were observed only in the men, which is in line with our previous cross-sectional findings based on the BASE-II baseline data [30]. The longitudinal findings for the men were independent of other putative confounding parameters that may have affected lung function (i.e., age, BMI, pack-years, regular alcohol intake, self-reported physical inactivity, and morbidity index). The results presented in this follow-up study reaffirm our previous findings [30], in which Lp(a) quintile 1 was cross-sectionally associated with poorer lung function in men. The current study further expands the existing knowledge based on cross-sectional data [30] by analyzing the decline in lung function over time. We observed a statistically significant decline in lung volumes (FEV1 and FVC) in both sexes after 7.4 years. In relation to the serum Lp(a) levels at the baseline, the decline in lung function was the greatest in the men with low Lp(a) concentrations. The men with the highest Lp(a) concentrations (Lp(a) quintile 5) showed the least decline in lung function after 7.4 years. In addition, it appears that the relationship between Lp(a) concentration and lung function is non-linear. When analyzing the mean FEV1 and FVC of the individual Lp(a) quintiles 1 to 5 in ascending order, the men in Lp(a) quintile 3 and quintile 4 showed the highest mean FEV1 and FVC values. The lung function measurements for each Lp(a) quintile from 2 to 4 were higher than those for Lp(a) quintile 1 and quintile 5, in both the men and women. While the underlying mechanism of this relationship remains unclear, this may indicate that a moderately high Lp(a) concentration, and perhaps not necessarily a very high Lp(a) concentration, could have a beneficial and possibly causal role in lung function. In the final step, we performed two-sample MR analyses to probe for a potential causal relationship between both traits using independent GWAS data from the UK biobank. Despite being sufficiently powered to detect such effects, none of these MR analyses suggested causal effects between Lp(a) and the lung function parameters investigated here (i.e., FEV1 and FVC). In addition to our previous study based on the cross-sectional BASE-II baseline data [26], there are only a few studies that have investigated the relationship between Lp(a) and measures related to pulmonary health, with rather inconsistent outcomes. Cardiorespiratory fitness, measured as peak oxygen uptake, was inversely associated with Lp(a) in a cohort of 425 Korean men with type 2 diabetes [44]. In a phenome-wide association study using individual-level data from 112,338 individuals of European ancestry from the UK Biobank, Emdin et al. found no association between genetically lowered Lp(a) and four types of respiratory disorders (i.e., asthma, COPD, pneumonia, and hay fever) [45]. Meanwhile, a study which assessed Lp(a) behavior in 90 COPD patients showed serum Lp(a) levels were significantly lower in COPD patients than in healthy subjects [46]. So far, there has only been one other study that has analyzed the relationship between Lp(a) and pulmonary function assessed by spirometry (FEV1 and FVC) in a population-based cohort. Contradictory to our findings, the cross-sectional study by Lee et al. observed an inverse association, in which reduced lung function was associated with elevated Lp(a) levels in a cohort of 64,082 Korean health-screening examinees [31]. Several factors could account for the incongruent results regarding the Lp(a)–lung function relationship. The mean age of the enrolled participants in the study by Lee and colleagues was 38 ± 7 years, and thereby considerably younger than the median age of the current study (68 [65–71] years at the baseline, 76 [73–78] years at the follow-up). The authors of this study speculate that due to the higher mean age, our cohort may have included more subjects with subclinical hepatic dysfunction, which could have affected the Lp(a) levels, as Lp(a) is synthesized exclusively by the liver [4,47]. Although age-related changes in hepatic structure and function have been described [48], hepatic function seems to be quite well maintained in old age [49]. Our study included generally healthy participants, with liver-related parameters, such as gamma-glutamyl transferase (GGT), alanine aminotransferase (ALT), and alkaline phosphatase (ALP), on average within the clinical normal range, at both the baseline and follow-up (Table 2) [50]. Furthermore, the observed association between Lp(a) quintile 1 and lower lung function measurements was statistically stable even after adjustments for GGT, ALT, and ALP (Supplementary Table S4). The results of these follow-up analyses suggest that an age-related reduction in liver function is unlikely to explain the observed relationship between Lp(a) and lung function. Notably, our cohort consists of participants of European ancestry, whereas the study by Lee et al. [31] consisted of Korean participants. Several studies have demonstrated ethnic differences in Lp(a) levels due to genetic variations across ethnic groups [51], which, at least in part, might explain the discrepancy in the results of our study and the study from Korea mentioned above.

Given that both impaired lung function and high Lp(a) levels are recognized risk factors for cardiovascular disease (CVD), the positive relationship between Lp(a) and lung function observed in our study appear inconsistent at first. However, several studies have also demonstrated rather unexpected relationships between Lp(a) and risk factors for CVD, which stand in marked contrast to prior studies that have shown a positive association of Lp(a) with CVD. Numerous studies, including the BASE-II, have reported low levels of Lp(a) being associated with a higher risk of T2D and MetS [43,52,53,54,55,56,57]. The association between low Lp(a) levels and lower lung function observed in the current study is in accordance with such findings, as both T2D and MetS are also known to be associated with decreased lung volumes [58,59]. In the BASE-II sub-cohort studied here, the prevalence of T2D was higher in Lp(a) quintile 1 than in Lp(a) quintiles 2-5 (*p* = 0.010), but there was no significant difference between the two groups regarding the prevalence of MetS (Table 4). Additional adjustments for T2D and MetS as covariables had no meaningful impact on the association between Lp(a) and lung function (Supplementary Table S4). The recalculation of the linear regression models after excluding subjects with T2D from the main cohort (n = 605) further showed a statistically significant association between Lp(a) quintile 1 and reduced FEV1 and FVC in the men, as well as in both sexes combined (Supplementary Tables S5 and S6). Onat and colleagues postulated that enhanced inflammation and autoimmune activation may result in reduced circulating Lp(a), due to the “trapping” of Lp(a) by immune complex formation, yielding a reduced assayed Lp(a) concentration [60]. According to this notion, low Lp(a) levels may have resulted from an inflammation-related mechanism associated with T2D, MetS, and lung function impairment.

The physiological function and exact mechanisms that underlie the role of Lp(a) in lung function remain unclear. Emerging studies regarding the role of cholesterol and lipoproteins in lung surfactant suggest a possible link between lipoprotein molecules and lung (patho)physiology [61,62,63,64,65,66,67,68,69]. Lipid tracer studies in rodents have estimated that more than 80% of surfactant cholesterol is derived from serum lipoproteins, highlighting the possibility that dyslipidemia in humans may dysregulate the lipid composition of lung surfactant, a vital component of healthy lung function. Furthermore, circulating lipoproteins, including high-density lipoprotein cholesterol (HDL-C), low-density lipoprotein cholesterol (LDL-C), and very low-density lipoprotein (VLDL), play a vital role in normal lung function, as they are known to affect both the synthesis and secretion of lung surfactant [70,71]. In a study by Barochia et al., HDL-C and apolipoprotein A1 (apoA-I) were positively correlated with FEV1, whereas LDL-C, triglycerides, apolipoprotein B (apoB), and the apoB/apoA-I ratio were negatively correlated with FEV1 among patients with atopic asthma [62]. Lastly, given that our MR results did not reveal evidence for a causal effect of Lp(a) on FEV1 and FVC, it appears that the observed relationship between these traits is more complex than anticipated, and the potential causal factor(s) linking these variables remain to be identified. The Lp(a)–lung function relationship observed in the women showed a similar pattern compared to that observed for the men, but there was no statistically supported difference in the lung function measurements between Lp(a) quintile 1 and Lp(a) quintiles 2-5 in the women. This “weaker” relationship in women compared to that in men was also observed in our previous study based on cross-sectional baseline data [30], and the reasons for the differing results are likely multifactorial. The effects of postmenopausal status on both Lp(a) [72,73,74,75] and lung function [76,77,78,79], as well as the sex-related differences regarding lung health [80,81], may at least partly explain why the Lp(a)–lung function association was not as evident in women as in men. However, this could also be a matter of statistical power, since the direction of the associations observed in men and women were quite similar (Figure 4).

The cross-sectional analyses using the re-measured Lp(a) levels from the follow-up showed a similar trend in the men regarding the relationship between Lp(a) quintile 1 and lung function, however, with no longer statistical significance. This loss of significance may be due to multifactorial reasons. Despite knowing that Lp(a) levels are predominantly genetically determined and cannot be easily modulated by life-style changes, there was a significant increase in the proportion of the men categorized in Lp(a) quintile 1 at the baseline versus at the follow-up (20% at the baseline vs. 25% at the follow-up, *p* = 0.010, Table 2), which may have affected our cross-sectional results. Other aging-associated changes over the 7.4 years, such as the significant increase in participants with T2D at the follow-up, may have further affected the cross-sectional relationship between Lp(a) and lung function, as it has been hypothesized that increased insulin levels, in the context of insulin resistance, reduce Lp(a) synthesis [82,83,84].

As a limitation of our study, we acknowledge a potential selection bias, since only those individuals who participated in both the baseline and follow-up assessments were included in our analysis, as well as only those with a spirometry quality grade of “C” or above were included in this study, further adding to a potential bias. Additionally, our study does not reflect the general population, as the cohort studied was limited to voluntarily participating residents of the Berlin metropolitan area, aged 60 years and older. However, the longitudinal aspect of this follow-up study was able to reaffirm and expand on the previously observed association between Lp(a) and lung function at the baseline. Our study has several strengths that should be acknowledged. This study’s size was relatively substantial, with a total number of 679 participants—greater than that of the first cross-sectional analysis of the BASE-II, as we obtained a higher number of sufficient quality spirometry data in the follow-up study. Furthermore, we obtained two separate Lp(a) measurements at the baseline and follow-up, with a span of 7.4 ± 1.5 years in between, which helped confirm our previous findings through longitudinal as well as cross-sectional analyses. To our knowledge, this is the first longitudinal study to assess the association between Lp(a) and lung function in older people.

Currently, there is high interest amongst clinicians and researchers in the development of a pharmacologic therapy that could lower Lp(a) levels in order to reduce the risk of cardiovascular diseases. A phase III trial evaluating an antisense oligonucleotide therapy is currently being awaited with great anticipation [19,20,21,22,23]. Although we did not find evidence of a causal relationship between Lp(a) and the lung function parameters assessed, it is important to keep in mind the observed relationship when evaluating the safety of new pharmacologic therapies designed to modify Lp(a) levels. Additionally, it would be desirable to see the results of other longitudinal studies and prospective interventional studies with a more diverse age group on the topic investigated here. We recommend that future studies take into consideration how biological sex might influence the association between Lp(a) and pulmonary function, as both Lp(a) (mainly for postmenopausal women) and lung function are influenced by sex-linked biological differences [85,86].

## 5. Conclusions

In conclusion, we observed that elevated Lp(a) levels at the baseline were associated with better lung function after an average follow-up time of 7.4 years in older men. Women showed similar findings, but the association between Lp(a) level and lung function was not statistically apparent. This is suggestive of elevated serum Lp(a) playing an unknown and possibly sex-specific beneficial role in pulmonary health. As indicated by our genetic analyses, the role of Lp(a) in the observed associations does not seem to be causal, based on the currently available GWAS data. Thus, future work needs to independently validate the association between Lp(a) and lung function and identify the potential causal factor(s) underlying this relationship. There are currently no studies that have examined the physiological role of Lp(a) in lung (patho)physiology in general. These large gaps in our understanding of the Lp(a)–lung function relationship point to directions for future research.

## Figures and Tables

**Table 1:** Characteristics of participants at baseline and lung function measurements at follow-up, 7.4 ± 1.5 years later.

Baseline	All (n = 679)	Men (n = 326)	Women (n = 353)	*p*-Value *
Age [years]	68 (65–71)	68 (65–71)	68 (65–70)	0.108
BMI [kg/m^2^]	26.2 (23.8–28.7)	26.9 (24.8–29.0)	25.1 (22.9–28.0)	<0.001
Current smoker [n; %]	61 (9)	30 (9)	31 (9)	0.894
Pack-years [years]	0.5 (0.0–15.0)	7 (0.0–22.2)	0 (0.0–6.1)	<0.001
Regular alcohol intake [n; %]	611 (90)	297 (91)	314 (89)	0.289
Self-reported physical inactivity [n; %]	55 (8)	33 (10)	22 (6)	0.066
Morbidity index [pts.]	1 (0–2)	1 (0–2)	0 (0–1)	0.052
T2D [n; %]	74 (11)	52 (16)	22 (6)	<0.001
MetS [n; %]	230 (34)	141 (44)	89 (26)	<0.001
Lipoprotein(a) [mg/dL]	10.6 (5.0–28.1)	9.0 (4.0–21.0)	12.0 (5.0–42.0)	0.002
**Follow-up**				
FEV1 [ml]	2337 ± 615	2737 ± 563	1967 ± 387	<0.001
FVC [ml]	3115 ± 798	3686 ± 671	2588 ± 480	<0.001
FEV1/FVC [%]	76 (72–79)	75 (71–79)	76 (73–80)	<0.001

Results are shown as mean ± SD for variables with normal distribution and as median (interquartile range) for variables with skewed distribution. * Student’s *t*-test for normally distributed data or Mann–Whitney U-Test for skewed data. * Categorical variables between two groups were compared using chi-square test or Fisher’s exact test.

**Table 2:** Characteristics of subjects with longitudinal data available at baseline and at follow-up (paired comparison).

	Men (n = 326)			Women (n = 353)			All (n = 679)		
	Baseline	Follow-Up	*p*-Value *	Baseline	Follow-Up	*p*-Value *	Baseline	Follow-Up	*p*-Value *
Age [years]	68 (65–71)	76 (72–78)	<0.001	68 (65–70)	76 (73–78)	<0.001	68 (65–71)	76 (73–78)	<0.001
BMI [kg/m^2^]	26.9 (24.8–29.0)	26.8 (24.7–29.1)	0.136	25.1 (22.9–28.0)	25.5 (23.0–28.7)	<0.001	26.2 (23.8–28.7)	26.3 (23.9–29.0)	<0.001
Current smoker [n; %]	30 (9)	16 (5)	0.001	31 (9)	20 (6)	0.003	61 (9)	36 (5)	<0.001
Pack-years [years]	7 (0.0–22.2)	6.3 (0.0–22-5)	0.853	0 (0.0–6.1)	0.0 (0.0–5–0)	0.270	0.5 (0.0–15.0)	0.0 (0.0–15.0)	0.423
Regular alcohol intake [n; %]	297 (91)	275 (84)	<0.001	314 (89)	288 (82)	<0.001	611 (90)	563 (83)	<0.001
Self-reported physical inactivity [n; %]	33 (10)	41 (13)	0.401	22 (6)	40 (11)	0.028	55 (8)	81 (12)	0.024
Morbidity index [pts.]	1 (0–2)	1 (0–2)	<0.001	0 (0–1)	1 (0–2)	<0.001	1 (0–1)	1 (0–2)	<0.001
T2D [n; %]	52 (16)	70 (21)	<0.001	22 (6)	42 (12)	<0.001	74 (11)	112 (16)	<0.001
GGT [U/L]	25 (19–37)	27 (20–37)	0.033	17 (14–25)	19 (15–28)	<0.001	21 (15–31)	23 (16–33)	<0.001
ALT [U/L]	22 (17–28)	21 (17–27)	0.559	18 (14–22)	18 (14–23)	0.104	19 (15–25)	20 (15–24)	0.496
AP [U/L]	58 (51–70)	N/A	N/A	63 (53–74)	N/A	N/A	60 (52–72)	N/A	NA
Lp(a) [mg/dL]	9.0 (4.0–21.0)	8.2 (3.4–19.0)	0.354	12.0 (5.8–42.0)	11.4 (5.5–43.1)	0.244	10.9 (5.0–28.3)	9.4 (5.3–28.2)	0.821
Lp(a) quintile 1 [n; %]	65 (20)	82 (25)	0.010	70 (20)	74 (21)	0.345	128 (19)	156 (23)	0.001

Results are shown as mean ± SD for variables with normal distribution and as median (interquartile range) for variables with skewed distribution. * Paired Student’s *t*-test (for normally distributed data), Wilcoxon signed-rank test (for skewed data), or McNemar’s test (for binominal data).

**Table 3:** Lung function measurements at baseline and follow-up (paired comparison).

	Men (n = 173)			Women (n = 183)		
	Baseline	Follow-Up	*p*-Value *	Baseline	Follow-Up	*p*-Value *
FEV1 [mL]	3064 ± 521	2733 ± 511	<0.001	2228 ± 418	1958 ± 404	<0.001
FVC [mL]	4195 ± 671	3707 ± 647	<0.001	2944 ± 516	2580 ± 479	<0.001
FEV1/FVC [%]	74 (69–78)	74 (70–78)	0.183	76 (72–80)	77 (73–80)	0.369

Results are shown as mean ±SD for variables with normal distribution and as median (interquartile range) for variables with skewed distribution. * Student’s *t*-test for normally distributed data or Mann–Whitney U-Test for skewed data.

**Table 4:** Lp(a) quintile 1 vs. 2-5: baseline characteristics of participants with lung function data available at follow-up.

	Men (n = 326)			Women (n = 353)			All		
Baseline	Lp(a) Quintile 1	Lp(a) Quintiles 2-5	*p*-Value *	Lp(a) Quintile 1	Lp(a) Quintiles 2-5	*p*-Value *	Lp(a) Quintile 1	Lp(a) Quintiles 2-5	*p*-Value *
Age [years]	67 (65–71)	69 (66–71)	0.288	68 (65–70)	68 (65–70)	0.205	68 (65–71)	68 (66–71)	0.066
BMI [kg/m^2^]	27.2 (24.5–29.5)	26.9 (24.9–29.0)	0.546	24.4 (23.0–28.1)	25.4 (22.8–27.9)	0.695	26.0 (23.8–28.7)	26.2 (23.8–28.7)	0.887
Current smoker [n; %]	9 (14)	21 (8)	0.136	7 (10)	24 (9)	0.906	14 (11)	47 (9)	0.383
Pack-years [years]	9.0 (0.0–33.8)	6.3 (0.0–20.0)	0.177	0.0 (0.0–7.5)	0.0 (0.0–6.0)	0.957	1.0 (0.0–19.3)	0.0 (0.0–14.0)	0.563
Regular alcohol intake [n; %]	57 (88)	240 (92)	0.460	65 (93)	249 (88)	0.244	115 (91)	496 (90)	0.856
Self-reported physical inactivity [n; %]	9 (14)	24 (9)	0.271	5 (7)	17 (6)	0.783	14 (11)	41 (8)	0.200
Morbidity index [pts.]	1 (0–2)	1 (0–2)	0.751	1 (0–1)	0 (0–1)	0.463	1 (0–2)	1 (0–1)	0.380
T2D [n; %]	15 (23)	37 (14)	0.082	8 (12)	14 (5)	0.041	23 (17)	51 (10)	0.010
MetS [n; %]	28 (43)	113 (44)	0.897	22 (32)	67 (24)	0.196	50 (37)	180 (34)	0.441
**Follow-up**									
FEV1 [mL]	2577 ± 570	2776 ± 555	0.010	1936 ± 380	1975 ± 390	0.461	2293 ± 576	2347 ± 624	0.372
FVC [mL]	3520 ± 630	3728 ± 676	0.026	2541 ± 461	2600 ± 484	0.359	3084 ± 731	3123 ± 813	0.623
FEV1/FVC [%]	74 (70–78)	75 (71–79)	0.140	77 (72–80)	76 (73–79)	0.729	76 (71–79)	76 (72–79)	0.335

Results are shown as mean ±SD for variables with normal distribution and as median (interquartile range) for variables with skewed distribution. * Student’s *t*-test for normally distributed data or Mann–Whitney U-Test for skewed data. * Categorical variables between two groups were compared using chi-square test or Fisher’s exact test.

**Table 5:** Multiple regression analyses: association between Lp(a) quintile 1 * at baseline and lung function at follow-up.

Lp(a) Quintile 1 *	Men									Women								
	FEV1			FVC			FEV1/FVC			FEV1			FVC			FEV1/FVC		
	Beta	SE	*p*	Beta	SE	*p*	Beta	SE	*p*	Beta	SE	*p*	Beta	SE	*p*	Beta	SE	*p*
Model 1	−225	74	0.003	−237	89	0.008	−17	9	0.078	−55	50	0.272	−78	62	0.205	−1	8	0.926
Model 2	−216	77	0.005	−231	92	0.013	−15	10	0.135	−69	50	0.166	−99	61	0.105	−1	8	0.923
Model 3	−218	81	0.008	−234	97	0.017	−15	10	0.156	−60	52	0.251	−87	64	0.175	−1	8	0.927

Model 1: adjusted for age. Model 2: model 1 + regular alcohol intake, self-reported physical inactivity, pack-years, and BMI. Model 3: model 2 + morbidity index. * Lp(a) quintile 1 and Lp(a) quintiles 2-5 used as binary variables.

## Data Availability

Due to concerns for participant privacy, data are available only upon reasonable request. Interested investigators are invited to contact the scientific coordinator of BASE-II, Ludmila Müller (lmueller@mpib-berlin.mpg.de). Additional information can be obtained from the BASE-II website: https://www.base2.mpg.de/7549/data-documentation (accessed on 11 June 2024).

## Supplementary Materials

The following supporting information can be downloaded at: https://www.mdpi.com/article/10.3390/biomedicines12071502/s1, Figure S1: Cross-sectional analysis - association between Lp(a) re-measured at follow-up and lung function measured at follow-up; Figure S2: Results of two-sample MR analyses using GWAS results on Lp(a) and lung function; Table S1: Multiple linear regression: relationship between Lp(a) quintile 1 at baseline and lung function at follow-up - adjusted for covariables from follow-up; Table S2: Characteristics of participants at follow-up according to Lp(a) quintile 1 vs quintiles 2-5 re-measured at follow-up – a cross-sectional analysis of follow-up study; Table S3: Multiple linear regression: relationship between Lp(a) quintile 1 and lung function both at follow-up - adjusted for covariables from follow-up; Table S4: Multiple linear regression: association between Lp(a) quintile 1 at baseline and lung function at follow-up - adjusted for covariables from baseline including type 2 diabetes mellitus, metabolic syndrome, and parameters of liver function; Table S5: Multiple linear regression: association between Lp(a) quintile 1 at baseline and lung function at follow-up - adjusted for covariables from baseline excluding subjects with T2D (n=605), men and women analyzed separately; Table S6: Multiple linear regression: association between Lp(a) quintile 1 at baseline and lung function at follow-up - adjusted for covariables from baseline excluding subjects with T2D (n=605), men and women analyzed together.
