# Supplementary material for: Lipoprotein(a) and Lung Function Are Associated in Older Adults: Longitudinal and Cross-Sectional Analyses
Source: Biomedicines. 2024 Jul 6;12(7):1502. doi: 10.3390/biomedicines12071502 (PMC11274407; doi:10.3390/biomedicines12071502)
Supplement: Supplementary file 1 [file biomedicines-12-01502-s001.zip › biomedicines-3032849-supplementary.pdf]

Supplementary Tables & Figures

Lipoprotein(a) and Lung Function are Associated in Older Adults: Longitudinal and Cross-Sectional Analyses

Chae Kyung Song[1], Olena Ohlei[2], Theresa Keller[3], Vera Regitz-Zagrosek[4, 5], Sarah Toepfer[1], Elisabeth Steinhagen-Thiessen[1], Lars Bertram[2, 6], Nikolaus Buchmann[7, *], Ilja Demuth[1, 8, *]

[1]Charité – Universitätsmedizin Berlin, corporate member of Freie Universität Berlin and Humboldt-Universität zu Berlin, Department of Endocrinology and Metabolic Diseases (including Division of Lipid Metabolism), Biology of Aging working group, Augustenburger Platz 1, 13353 Berlin, Germany

[2]Lübeck Interdisciplinary Platform for Genome Analytics (LIGA), University of Lübeck, Lübeck, Germany

[3]Institute of Biometry and Clinical Epidemiology, Charité - Universitätsmedizin Berlin, Corporate Member of Freie Universität Berlin, Humboldt-Universität zu Berlin, and Berlin Institute of Health, Reinhardtstraße 58, 10117, Berlin, Germany.

[4]Institute for Gender in Medicine, Center for Cardiovascular Research, Charité - Universitätsmedizin Berlin, Corporate Member of Freie Universität Berlin, Humboldt - Universität zu Berlin and Berlin Institute of Health, Berlin, Germany

[5]Department of Cardiology, University Hospital Zürich, University of Zürich, Zürich, Switzerland

[6]Centre for Lifespan Changes in Brain and Cognition, University of Oslo, Oslo, Norway[7]Department of Cardiology, Charité - University Medicine Berlin (Campus Benjamin Franklin), Berlin, Germany.

[7]Department of Cardiology, Charité - University Medicine Berlin (Campus Benjamin Franklin), Berlin, Germany.

[8]Charité - Universitätsmedizin Berlin, BCRT - Berlin Institute of Health Center for Regenerative Therapies, Berlin, Germany

[*]joint last authors

Supplementary Table S1. Multiple linear regression: relationship between Lp(a) quintile 1 at baseline and lung function at follow-up - adjusted for covariables from follow-up

Lp(a) quintile 1*	Men									Women								
	FEV1			FVC			FEV1/FVC			FEV1			FVC			FEV1/FVC		
	Beta	SE	p	Beta	SE	p	Beta	SE	p	Beta	SE	p	Beta	SE	p	Beta	SE	p
Model 1	-218	73	0.003	-229	87	0.009	-16	9	0.088	-56	49	0.259	-80	61	0.190	-1	8	0.941
Model 2	-209	75	0.006	-230	88	0.010	-13	10	0.159	-54	49	0.277	-72	60	0.231	-2	8	0.776
Model 3	-239	81	0.003	-255	94	0.007	-16	10	0.129	-48	51	0.355	-76	63	0.225	2	8	0.826

Model 1: adjusted for age
Model 2: Model 1 + regular alcohol intake, self-reported physical inactivity, pack-years, BMI
Model 3: Model 2 + morbidity index
*Lp(a) quintile 1 and Lp(a) quintiles 2-5 used as binary variables

Supplementary Table S2. Characteristics of participants at follow-up according to Lp(a) quintile 1 vs quintiles 2-5 re-measured at follow-up – a cross-sectional analysis of follow-up study

	Male (n=326)			Female (n=351*)			All (n=677)		
Follow-up	Lp(a) quintile 1 (n=82)	Lp(a) quintile 2-5 (n=244)	p-value	Lp(a) quintile 1 (n=74)	Lp(a) quintile 2-5 (n=277)	p-value	Lp(a) quintile 1 (n=156)	Lp(a) quintile 2-5 (n=521)	p-value
Age [years]	75 (72-78)	76 (72-78)	0.711	75 (72-78)	76 (73-78)	0.034	75 (72-78)	76 (73-78)	0.135
BMI [kg/m2]	27.0 (24.4-29.4)	26.7 (24.8-29.0)	0.717	24.9 (22.9-29.6)	25.7 (22.9-28.5)	0.773	26.1 (24.0-29.4)	26.3 (23.8-28.7)	0.827
Pack-years [years]	8.0 (0.0-31.0)	5.9 (0.0-20-0)	0.500	0.0 (0.0-10.0)	0.0 (0.0-4.3)	0.354	0.1 (0.0-20.0)	0.0 (0.0-12.5)	0.291
Regular alcohol intake [n;%]	62 (76)	213 (87)	0.012	59 (80)	227 (82)	0.662	121 (78)	440 (85)	0.045
Self-reported physical inactivity [n;%]	6 (7)	35 (14)	0.095	7 (10)	32 (12)	0.604	13 (8)	67 (13)	0.121
Morbidity index [pts.]	2 (0-3)	1 (0-2)	0.090	1 (0-2)	1 (0-2)	0.719	1 (0-2)	1 (0-2)	0,297
Follow-up									
FEV1 [ml]	2633±575	2771±556	0.055	1972±411	1967±383	0.926	2319±602	2344±619	0.667
FVC [ml]	3601±658	3715±674	0.186	2585±496	2589±478	0.947	3119±775	3116±806	0.968
FEV1/FVC [%]	74 (70-77)	76 (71-79)	0.035	76 (73-80)	77 (73-80)	0.908	75 (71-78)	76 (72-79)	0.090

*2 participants excluded due to missing Lp(a) at follow-up

Supplementary Table S3. Multiple linear regression: relationship between Lp(a) quintile 1 and lung function both at follow-up - adjusted for covariables from follow-up

Lp(a) quintile 1*	Men									Women								
	FEV1			FVC			FEV1/FVC			FEV1			FVC			FEV1/FVC		
	Beta	SE	p	Beta	SE	p	Beta	SE	P	Beta	SE	p	Beta	SE	p	Beta	SE	P
Model 1	-141	67	0.038	-117	81	0.148	-17	9	0.048	-23	48	0.636	-38	60	0.524	-1	8	0.848
Model 2	-111	71	0.119	-84	83	0.315	-15	9	0.098	-26	48	0.587	-41	59	0.494	-2	8	0.759
Model 3	-107	77	0.168	-70	90	0.438	-16	10	0.097	-55	52	0.291	-86	63	0.174	0	8	0.967

Model 1: adjusted for age
Model 2: Model 1 + regular alcohol intake, self-reported physical inactivity, pack-years, BMI
Model 3: Model 2 + morbidity index
*Lp(a) quintile 1 and Lp(a) quintiles 2-5 used as binary variables

Supplementary Table S4. Multiple linear regression: association between Lp(a) quintile 1 at baseline and lung function at follow-up - adjusted for covariables from baseline including type 2 diabetes mellitus, metabolic syndrome, and parameters of liver function

Lp(a) quintile 1*	Men									Women								
	FEV1			FVC			FEV1/FVC			FEV1			FVC			FEV1/FVC		
	Beta	SE	p	Beta	SE	p	Beta	SE	p	Beta	SE	p	Beta	SE	p	Beta	SE	p
Model 1	-225	74	0.003	-237	89	0.008	-17	9	0.078	-55	50	0.272	-78	62	0.205	-1	8	0.926
Model 2	-216	77	0.005	-231	92	0.013	-15	9	0.135	-69	50	0.166	-99	61	0.105	-1	8	0.923
Model 3	-218	81	0.008	-234	97	0.017	-15	10	0.156	-60	52	0.251	-87	64	0.175	1	8	0.927
Model 4	-219	82	0.008	-234	98	0.017	-15	10	0.149	-60	52	0.249	-85	64	0.190	-2	8	0835
Model 5	-227	82	0.006	-241	98	0.014	-15	10	0.134	-64	52	0.225	-90	65	0.165	-2	8	0.850
Model 6	-209	83	0.013	-210	99	0.035	-16	11	0.125	-56	53	0.289	-86	66	0.191	-1	8	0.900

Model 1: adjusted for age
Model 2: Model 1 + regular alcohol intake, self-reported physical inactivity, pack-years, BMI
Model 3: Model 2 + morbidity index
Model 4: Model 2 + morbidity index without T2D + T2D
Model 5: Model 4 + MetS
Model 6: Model 5 + GGT, ALT, AP
*Lp(a) quintile 1 and Lp(a) quintiles 2-5 used as binary variables

Supplementary Table S5. Multiple linear regression: association between Lp(a) quintile 1 at baseline and lung function at follow-up - adjusted for covariables from baseline excluding subjects with T2D (n=605), men and women analyzed separately

Lp(a) quintile 1*	Men									Women								
	FEV1			FVC			FEV1/FVC			FEV1			FVC			FEV1/FVC		
	Beta	SE	p	Beta	SE	p	Beta	SE	p	Beta	SE	p	Beta	SE	p	Beta	SE	p
Model 1	-224	84	0.008	-207	99	0.038	-24	11	0.032	-54	53	0.310	-72	66	0.274	-2	8	0.780
Model 2	-221	88	0.013	-222	104	0.033	-19	11	0.097	-71	53	0.175	-100	65	0.121	-1	8	0.875
Model 3	-231	94	0.014	-231	110	0.037	-20	12	0.099	-62	55	0.263	-87	68	0.204	-2	9	0.856

Model 1: adjusted for age
Model 2: Model 1 + regular alcohol intake, self-reported physical inactivity, pack-years, BMI
Model 3: Model 2 + morbidity index
*Lp(a) quintile 1 and Lp(a) quintiles 2-5 used as binary variables

Supplementary Table S6. Multiple linear regression: association between Lp(a) quintile 1 at baseline and lung function at follow-up - adjusted for covariables from baseline excluding subjects with T2D (n=605), men and women analyzed together

Lp(a) quintile 1*	All (n = 605)								
	FEV1			FVC			FEV1/FVC		
	Beta	SE	p	Beta	SE	p	Beta	SE	p
Model 1	-132	63	0.038	-136	82	0.100	-12	7	0.083
Model 2	-159	65	0.016	-185	84	0.029	-8	7	0.247
Model 3	-172	69	0.013	-200	89	0.025	-9	7	0.227

Supplementary Figure S1. Cross-sectional analysis - association between Lp(a) re-measured at follow-up and lung function measured at follow-up. Distributions of FEV1 (A) and FVC (B) measurements are compared between participants belonging to Lp(a) quintile 1 and Lp(a) quintiles 2-5 separately for men and women. Mean FEV1 and FVC measurements were higher in men categorized in Lp(a) quintiles 2-5 than those from Lp(a) quintile 1. In women there was also no meaningful association between Lp(a) and lung function measurements both measured at follow-up.

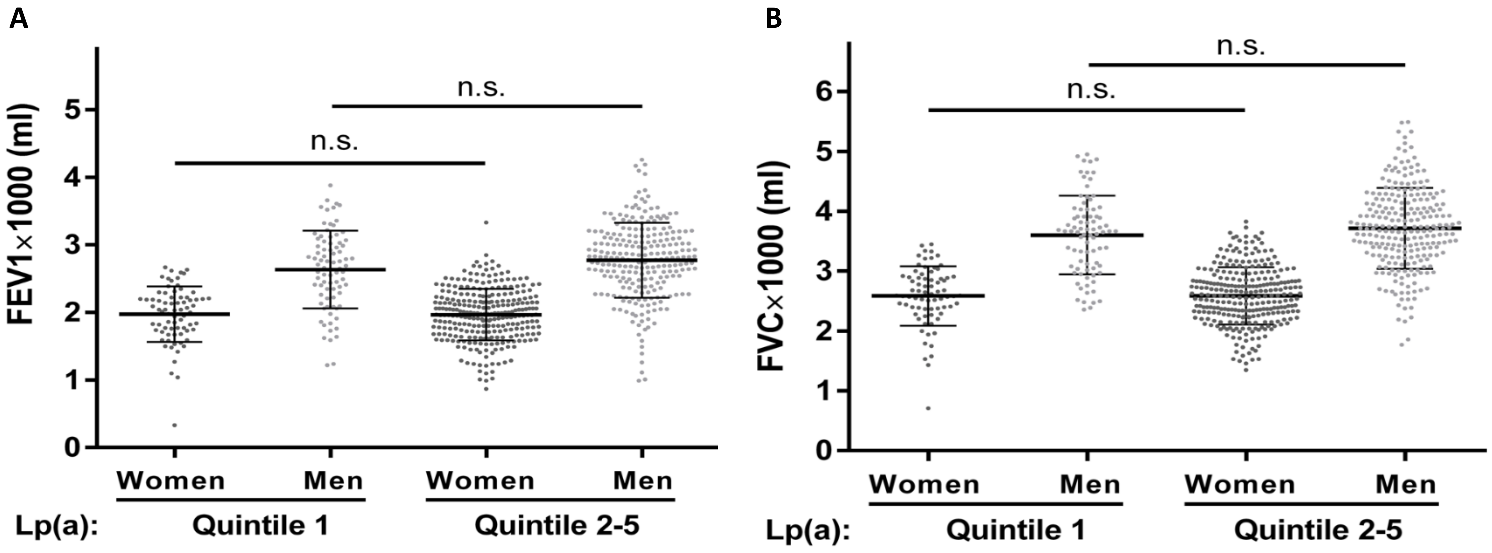

Supplementary Figure S2. Results of two-sample MR analyses using GWAS results on Lp(a) and lung function. Colored lines represent causal estimates from the different methods. A) FVC, B) FEV1.

A. FVC

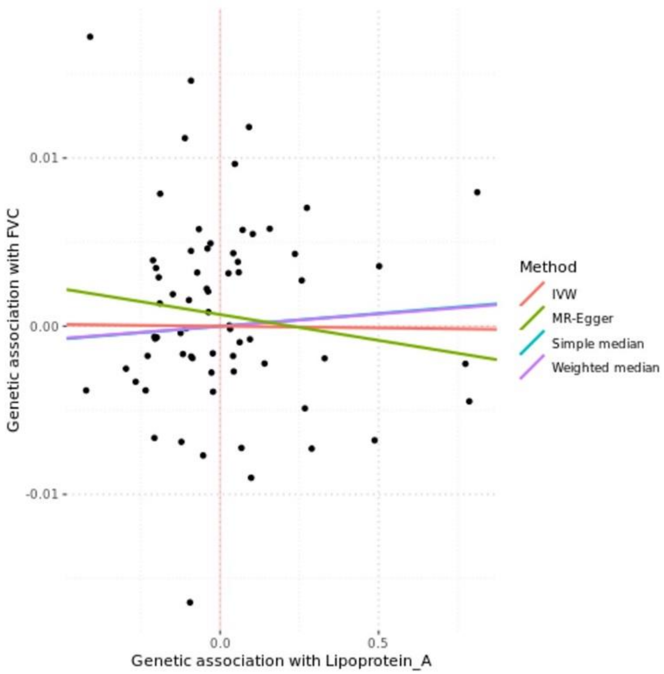

Method	Estimate	SE	95% CI	95% CI	P-value
Simple	0.002	0.006	-0.009	0.012	0.783
Weighted	0.001	0.004	-0.007	0.01	0.744
IVW	0	0.003	-0.006	0.006	0.946
MR-Egger	-0.003	0.004	-0.011	0.005	0.468
(intercept)	0.001	0.001	-0.001	0.002	0.311

B. FEV1

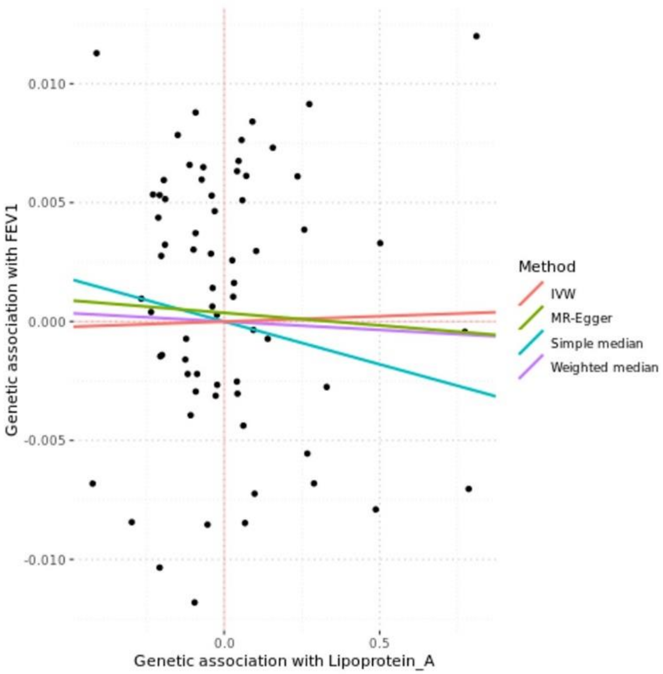

Method	Estimate	SE	95% CI	95% CI	P-value
Simple	-0.004	0.006	-0.015	0.008	0.552
Weighted	-0.001	0.005	-0.01	0.009	0.884
IVW	0	0.003	-0.006	0.007	0.89
MR-Egger	-0.001	0.004	-0.01	0.008	0.809
(intercept)	0	0.001	-0.001	0.002	0.606
